# Whole-genome sequencing and assembly of fish pathogenic *Proteus mirabilis* (COFI-RLBCAU-II) possessing multiple antimicrobial resistance genes

**DOI:** 10.1128/mra.00546-25

**Published:** 2025-09-26

**Authors:** Partha Sarathi Tripathy, Satya Narayan Parida, Anuj Tyagi, Ajaya Kumar Rout, Bijay Kumar Behera, Pramod Kumar Pandey

**Affiliations:** 1 College of Fisheries (Datia), Rani Lakshmi Bai Central Agricultural University 560178, Jhansi, Uttar Pradesh, India; 2 National Fisheries Development Board, Department of Fisheries, Government of India 601675 , Hyderabad, Telangana, India; The University of Arizona, Tucson, Arizona, USA

**Keywords:** fish pathogens

## Abstract

The study reports a fish pathogenic and multidrug-resistant *Proteus mirabilis* COFI-RLBCAU-II (CP181371) isolated from diseased *Labeo catla*. The bacterium belonged to the *Enterobacteriaceae* family, and its genome length was 3.93 Mb with 3,656 genes.

## ANNOUNCEMENT

*Proteus mirabilis* is a Gram-negative, rod-shaped, facultative anaerobic bacterium. It has been reported as lethal to the Indian major carp (1). *P. mirabilis* also has zoonotic potential, with the capability to cause urinary system infections in humans (2). In the present study, *P. mirabilis* was isolated from diseased *Labeo catla* with clinical signs like gross lesions and hemorrhage from a culture pond in Datia district (Madhya Pradesh, India) during March 2024. One gram of liver tissue was macerated and inoculated aseptically into sterile Tryptone Soy Broth (TSB) (3). After overnight incubation at 37°C, the inocula were streaked on Tryptone Soy Agar (TSA) plates, and morphologically distinct colonies were picked and subcultured once in Brain Heart Infusion (BHI) broth. The bacterial isolate up to genus *Proteus* was confirmed with biochemical assays and 16S rRNA gene sequencing (4). The same culture was used for WGS.

Prior to WGS of COFI-RLBCAU-II, a single colony from the pure culture was inoculated into sterile TSB at 37°C for 48 h with continuous agitation at 120 RPM. Thereafter, the bacterial genomic DNA was extracted following the protocol of Sambrook and Russell (5). The quality of DNA was assessed using a NanoDrop Spectrophotometer and a Qubit 3.0 Fluorometer (Thermo Fisher Scientific, USA). The NEBNext Companion Module Ligation Sequencing Kit (NEB, USA) was used for DNA end repair, and the library was prepared using the Native Barcoding Kit 24 V14 (SQK-NBD114.24) (Nanopore, UK). After QC, the concentration of the library was set to 50 fmol, and the library was loaded into the PromethION Flow cell (R10.4.1) (Nanopore, UK). *De novo* WGS was performed on the PromethION P2 Solo sequencer (Nanopore, UK) with an option to filter reads above 1 kb. The raw sequencing data were basecalled using Dorado v0.9.6 with a super accuracy model (dna_r10.4.1_e8.2_400bps_sup@v4.3.0) with default parameters, along with quality control and adapter trimming. The total number of raw reads after quality trimming was 8,290,781. Flye assembler v2.9.5 was used to assemble the genome with default parameters (6), and CheckM (7) to evaluate the completeness. The total number of contigs after the final assembly was found to be 4. The Prokaryotic Genome Annotation Process (PGAP) (8) was used to annotate the genome. The Bacterial and Viral Bioinformatics Resource Center (BV-BRC) (9) was used to analyze the antimicrobial resistance gene and virulence factors. A whole genome phylogeny was constructed using GToTree v1.8.13 (10) with default parameters. iTOL v6 (11) was used to design the phylogenomic tree.

**Fig 1:**
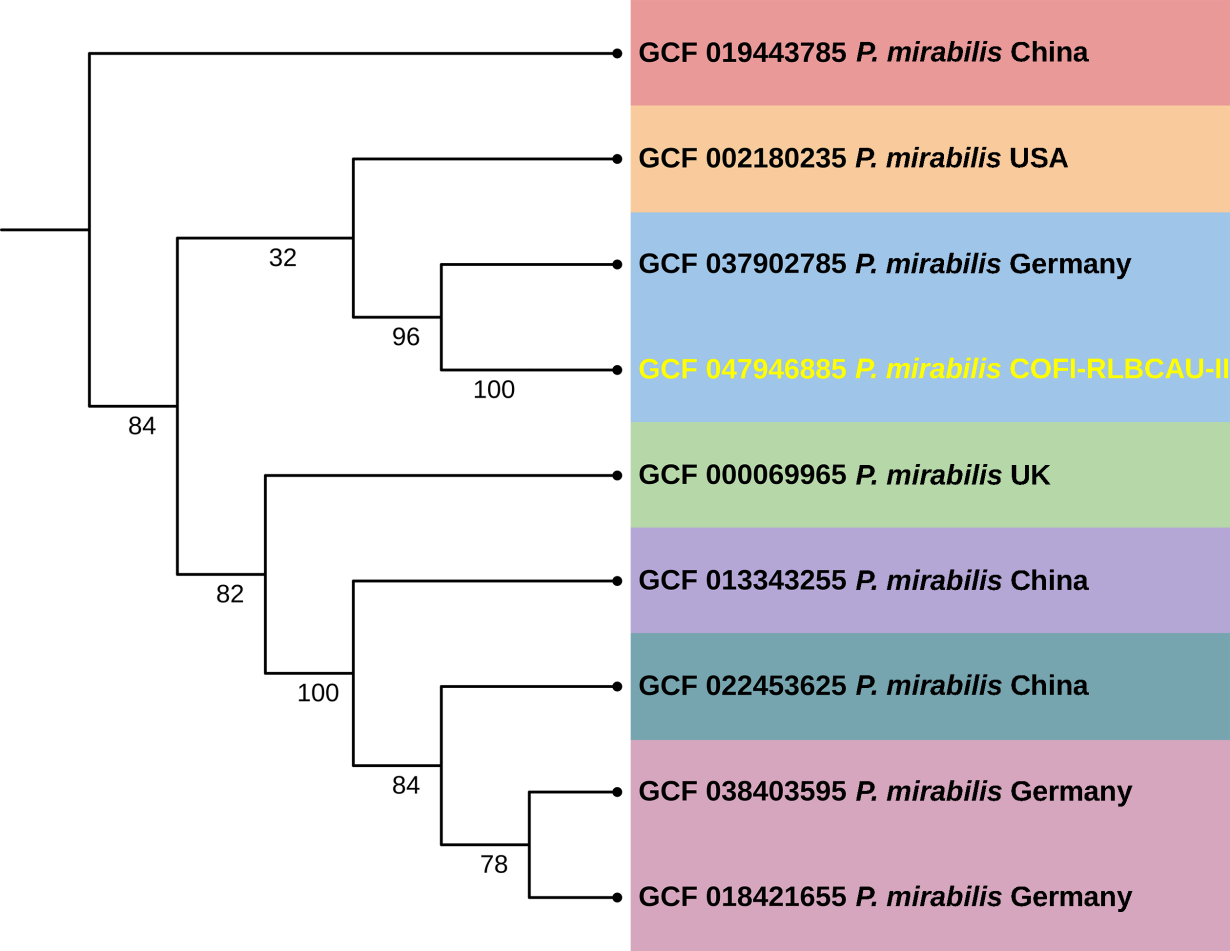
The phylogenomic tree contains the isolated *Proteus mirabilis* COFI-RLBCAU-II from this fish and other strains from various geographical locations. The evolutionary significance was generated based on WGS using GToTree based on 100 bootstrap replications, and iTol was used to depict the tree.

The genomic assembly features of *P. mirabilis* COFI-RLBCAU-II (CP181371), along with three plasmids, are given in Table 1. The study revealed the presence of AMR genes, such as *cat*, *murA*, *sat-1*, *CRP*, *GlpT*, *cpxR*, *rpoB*, *aadA*, *dfrA1*, *gyrB*, *parE*, and *tetj* with the potential to confer resistance against various antibiotics, viz., chloramphenicol, fosfomycin, streptothricin, carbapenems, tetracyclines, and aminoglycosides. The analysis also revealed the presence of 11 virulence-related genes. The isolated strain is closely related to other strains isolated from a different host and forms a close clade (Fig. 1).

**TABLE 1:** Information on complete genomic features of *Proteus mirabilis* COFI-RLBCAU-II isolated from *Labeo catla*

Bacterial isolate	*Proteus mirabilis* COFI-RLBCAU-II
Sequencing technology	Oxford Nanopore PromethION
Basecaller	Dorado v0.9.6
Assembly method	Flye v. 2.9.5
Genome coverage	1737 X
Total genome components	Chromosome – 1 Plasmids – 3
Genome length (bp)	Total: 3,979,959 (4 contigs) Chromosome: 3,933,471 (Circular) Plasmid 1: 3,866 (Circular) Plasmid 2: 17,385 (Circular) Plasmid 3: 25,237 (Circular)
GC content	38.661503
L50 of raw reads	822,409
N50 of raw reads	2,096
tRNA	90
rRNA	22
Protein coding genes	3,491
Antimicrobial resistance genes (total)	14
No. of plasmid	3 Plasmid 1: NZ_CP181372 Plasmid 2: NZ_CP181373 Plasmid 3: NZ_CP181374
BioProject number	PRJNA1207781
Biosample accession no.	SAMN46143986
GenBank accession number	GCF047946885

## Data Availability

The WGS sequence of Proteus mirabilis COFI-RLBCAU-II can be accessed from NCBI GenBank ID GCF047946885, and three plasmids (NZ_CP181372, NZ_CP181373, NZ_CP181374) can be accessed from NCBI. The raw sequence data are available in the Sequence Read Archive (SRA) database under accession numbers SRR34492528.

## References

[1] Pattanayak S, Kumar PR, Sahoo MK, Paul A, Sahoo PK. 2018. First field-based evidence of association of Proteus mirabilis causing large scale mortality in Indian major carp farming. Aquaculture 495:435–442. doi:10.1016/j.aquaculture.2018.06.006

[2] Chakkour M, Hammoud Z, Farhat S, El Roz A, Ezzeddine Z, Ghssein G. 2024. Overview of Proteus mirabilis pathogenicity and virulence. Insights into the role of metals. Front Microbiol 15:1383618. doi:10.3389/fmicb.2024.1383618 38646633 PMC11026637

[3] Kumar V, Das BK, Swain HS, Chowdhury H, Roy S, Bera AK, Das R, Parida SN, Dhar S, Jana AK, Behera BK. 2022. Outbreak of Ichthyophthirius multifiliis associated with Aeromonas hydrophila in Pangasianodon hypophthalmus: the role of turmeric oil in enhancing immunity and inducing resistance against co-infection. Front Immunol 13:956478. doi:10.3389/fimmu.2022.956478 36119096 PMC9478419

[4] Behera BK, Parida SN, Kumar VS, Swain HS, Parida PK, Bisai K, Dhar S, Das BK. 2023. Aeromonas veronii is a lethal pathogen isolated from gut of infected Labeo rohita: molecular insight to understand the bacterial virulence and its induced host immunity. Pathogens 12:598. doi:10.3390/pathogens12040598 37111485 PMC10143776

[5] Sambrook J, Russell DW. 2001. Molecular cloning a laboratory manual. 3rd ed. Vol. 1. Cold Spring Harbor Laboratory Press, New York.

[6] Kolmogorov M, Yuan J, Lin Y, Pevzner PA. 2019. Assembly of long, error-prone reads using repeat graphs. Nat Biotechnol 37:540–546. doi:10.1038/s41587-019-0072-8 30936562

[7] Parks DH, Imelfort M, Skennerton CT, Hugenholtz P, Tyson GW. 2015. CheckM: assessing the quality of microbial genomes recovered from isolates, single cells, and metagenomes. Genome Res 25:1043–1055. doi:10.1101/gr.186072.114 25977477 PMC4484387

[8] Tatusova T, DiCuccio M, Badretdin A, Chetvernin V, Nawrocki EP, Zaslavsky L, Lomsadze A, Pruitt KD, Borodovsky M, Ostell J. 2016. NCBI prokaryotic genome annotation pipeline. Nucleic Acids Res 44:6614–6624. doi:10.1093/nar/gkw569 27342282 PMC5001611

[9] Olson RD, Assaf R, Brettin T, Conrad N, Cucinell C, Davis JJ, Dempsey DM, Dickerman A, Dietrich EM, Kenyon RW, et al.. 2023. Introducing the bacterial and viral bioinformatics resource center (BV-BRC): a resource combining PATRIC, IRD and ViPR. Nucleic Acids Res 51:D678–D689. doi:10.1093/nar/gkac1003 36350631 PMC9825582

[10] Lee MD. 2019. GToTree: a user-friendly workflow for phylogenomics. Bioinformatics 35:4162–4164. doi:10.1093/bioinformatics/btz188 30865266 PMC6792077

[11] Letunic I, Bork P. 2024. Interactive Tree of Life (iTOL) v6: recent updates to the phylogenetic tree display and annotation tool. Nucleic Acids Res 52:W78–W82. doi:10.1093/nar/gkae268 38613393 PMC11223838

